# Salivary Metabolomics Fingerprint of Chronic Apical Abscess with Sinus Tract: A Pilot Study

**DOI:** 10.1155/2019/3162063

**Published:** 2019-11-16

**Authors:** Noemi Montis, Elisabetta Cotti, Antonio Noto, Claudia Fattuoni, Luigi Barberini

**Affiliations:** ^1^Department of Conservative Dentistry and Endodontics, University of Cagliari, Cagliari, Italy; ^2^Department of Medical Sciences and Public Health, University of Cagliari, Cagliari, Italy; ^3^Department of Chemical and Geological Sciences, University of Cagliari, Cagliari, Italy

## Abstract

Chronic apical abscess (CAA) is a lesion of apical periodontitis mostly characterized by areas of liquefactive necrosis with disintegrating polymorphonuclear neutrophils surrounded by macrophages. Its presence leads to local bacterial infection, systemic inflammatory response, pain, and swelling. The use of a novel approach for the study of CAA, such as metabolomics, seems to be important since it has proved to be a powerful tool for biomarkers discovery which could give novel molecular insight on CAA. So, the aim of this study was to verify the possibility to identify the metabolic fingerprint of CAA through the analysis of saliva samples. Nineteen patients were selected for this study: eleven patients affected by CAA with a sinus tract constituted the study group whereas eight patients without clinical and radiographic signs of CAA formed the healthy control group. Saliva samples were collected from each subject and immediately frozen at −80°C. Metabolomic profiles were obtained using a gas chromatography/mass spectrometry instrument. Subsequently, in order to compare the two groups, a multivariate statistical model was built that resulted to be statistically significant. The class of metabolites characterizing the CAA patients was closely related to the bacterial catabolism, tissue necrosis, and presence of a sinus tract. These preliminary results, for the first time, indicate that saliva samples analyzed by means of GC/MS metabolomics may be useful for identifying the presence of CAA, leading to new insights into this disease.

## 1. Introduction

Apical periodontitis (AP) is a pathologic condition of the oral periradicular tissues generated as a consequence of the inflammatory response at the root apices of teeth with necrotic infected pulps. Endodontic infections are usually restricted to the root canal system and include microorganisms, viruses, fungi, and yeasts [1–4]. The inflammatory response is believed to be an attempt to prevent the spread of the infection to the bone and to the entire system [5]. However, in some cases, microorganisms from the root canal may extend to the periradicular tissues and cause an *extraradicular* infection usually associated with symptoms and/or persistent apical periodontitis [6, 7]. Chronic apical abscess (CAA) is a lesion of AP which is mostly characterized by areas of liquefactive necrosis with disintegrating polymorphonuclear neutrophils (PMNs) surrounded by macrophages and normal PMNs [8]. This condition is associated with the intermittent drainage of the purulent content via a sinus tract, which opens in the oral mucosa (intraoral) or, less frequently, in the skin (extraoral) [5,6,9–11]. Sinus tracts in most cases exhibit an epithelial lining at the stoma, while its pathway may show an epithelial coating or be lined by an inflamed connective tissue [11, 12]. CAA has a prevalence of 8.5%–18%, in AP it is usually asymptomatic, often associated with larger, long-standing lesions (>5 mm in diameter) and an extraradicular infection [1, 7, 12–16]. Interestingly, the presence of a CAA with a sinus tract is considered a negative prognostic factor in the outcome of primary and secondary endodontic treatments; therefore, the use of novel approaches for the study of CAA seems important.

Metabolomics is one of the most recent research technologies focused on the quantitative and qualitative analyses of low-molecular weight metabolites (less than 1500 Daltons) present in biological fluids in response to a pathophysiological status [17]. It creates an instant snapshot of the biological status of a given organism, representing a sensitive measure of the biological status in health or disease [18]. The identification of an altered metabolic fingerprints offers novel opportunities to better understand physiological state, detect or identify potential risks for various diseases, and ultimately help achieve the goal of “personalized medicine.” It has been recently hypothesized that the presence of CAA can permanently modify the saliva metabolic content; therefore, it represents an important area for the aforementioned interventions [19]. This study aimed to identify the metabolic fingerprint of CAA patients through the metabolomic analysis of saliva and to open a novel insight into chronic apical periodontitis.

## 2. Materials and Methods

### 2.1. Clinical Population

This study was conducted on men and women observed at the Dental Clinic of the University of Cagliari, Italy, and was approved by the ethics committee. Following the recording of the medical and dental history, all patients received an accurate dental evaluation. A panoramic radiograph was performed as initial screening followed by intraoral radiographs taken on selected areas. Clinical examination was then performed on each individual and included the evaluation of hard and soft tissues (the presence and location of swelling and sinus tracts), confirming diagnosis of CAA by following sensitivity tests, cold test, palpation, percussion, and periodontal probing. Intraoral periapical radiographs were taken on all suspect teeth and all teeth were affected by CAA, tracing the sinus tract with a gutta-percha cone. The patients were then assigned to two groups according to the inclusion and exclusion criteria. Inclusion criteria were as follows: the presence of at least one tooth affected by CAA with a sinus tract, as assessed clinically and radiographically; good oral hygiene; presence of at least 20 teeth; absence of signs and symptoms of periodontal disease; absence of any known systemic disease; absence of pharmacological therapy; a range between 30 and 60 years of age. Exclusion criteria were as follows: the presence of poor oral hygiene; presence of localized or diffuse, chronic, or aggressive periodontitis; presence of destroying caries; patients who underwent chemotherapy or radiant therapy; patients who have been subjected to surgical dental procedures less than 15 days earlier (extraction, implant, periodontal, or endodontic therapies); patients under orthodontic therapy.

Nineteen subjects were divided into two groups. All subjects signed a written informed consent. Eleven patients affected by CAA formed the study group (group 1, average: 47 years old; standard deviation: 11.7 years old), while 8 patients free from clinical and radiographic evidence of CAA or AP were the healthy controls (group 2, average: 43.2 years old; standard deviation: 10.8 years old). Patients had similar demographic data (race, sex, and age distribution) (Table 1).

### 2.2. Metabolomic Analysis

Saliva samples (2 mL) were collected from each subject and immediately frozen at −80°C. The samples were subsequently thawed at room temperature, and 1 mL of saliva was transferred in an Eppendorf tube with 1 mL of cold acetone (−20°C), vortexed for 30 s, and centrifuged (14000 rpm at 4°C for 10 min) to remove the proteins [20]. 1 mL of supernatant was then transferred into glass vials and evaporated to dryness overnight in an Eppendorf vacuum centrifuge (Eppendorf AG, Hamburg, Germany). 30 *μ*L of a 0.24 M solution of methoxylamine hydrochloride in pyridine was added to each vial, and samples were vortex mixed and left to react for 17 h at room temperature. In the following step, 30 *μ*L of MSTFA (*N*-methyl-*N*-trimethylsilyltrifluoroacetamide) was added to the vials and left to react for 1 h at room temperature. Finally, the derivatized samples were diluted with hexane (600 *μ*L) and tetracosane (0.01 mg/ml) as internal standard, just before GC-MS analysis. Samples were analyzed using an Agilent 5977B interfaced to the GC 7890B (Agilent Technologies, Palo Alto, CA, USA), equipped with a DB-5ms column (Agilent J & W Scientific, Folsom, CA, USA), injector temperature at 230°C, detector temperature at 280°C, and helium carrier gas flow rate of 1 mL/min. The GC oven temperature program was set at 90°C initial temperature with 1 min hold time and ramping at 10°C/min to a final temperature of 270°C, with 7 min hold time. 1 *μ*L of the derivatized sample was injected in the split (1 : 20) mode. After a solvent delay of 3 minutes, mass spectra were acquired in full scan mode, using 2.28 scans/s with a mass range of 50–700 amu.

All acquired chromatograms were analyzed using the free software AMDIS (Automated Mass Spectral Deconvolution and Identification System, http://chemdata.nist.gov/mass-spc/amdis). Each chromatographic peak was identified by comparing the relative mass spectrum and retention time with those stored in an in-house made library including 234 metabolites. Other metabolites were identified using NIST08 (the National Institute of Standards and Technology's mass spectral database) and Golm Metabolome Database (GMD, http://gmd.mpimp-golm.mpg.de/). The metabolite was considered positively identified with a match factor ≥70%.

This strategy allowed for the detection of 76 compounds: 70 accurately identified and 6 unknown molecules recurring in every sample. AMDIS analysis produced an electronic sheet data matrix (Microsoft® Excel®, Microsoft Co, Redmond Washington DC, USA) that was submitted to multivariate statistical analysis. Data matrix was analyzed by the multivariate statistical analysis method, called orthogonal partial least square discriminant analysis (OPLS-DA), to obtain the set of important variables (metabolites) between the groups (SIMCA-P+ program, Version 13.0, Umetrics, Umea, Sweden). Subsequently, as metabolites are associated with biological functions, a pathway analysis was performed to discover a functional interpretation. The resulting “metabolome view” depicted all metabolic pathways arranged according to the scores from the enrichment analysis (*y*-axis) and from topology analysis (*x*-axis). Since many pathways were tested simultaneously, *p* values were adjusted for multiple testing. The goal of the pathway analysis was to identify the pathways which have a significant impact in a given phenotype and to verify whether the most important pathway modification resulted was affected by the periapical lesions and the sinus tract [21].

## 3. Results

**Figure 1:**
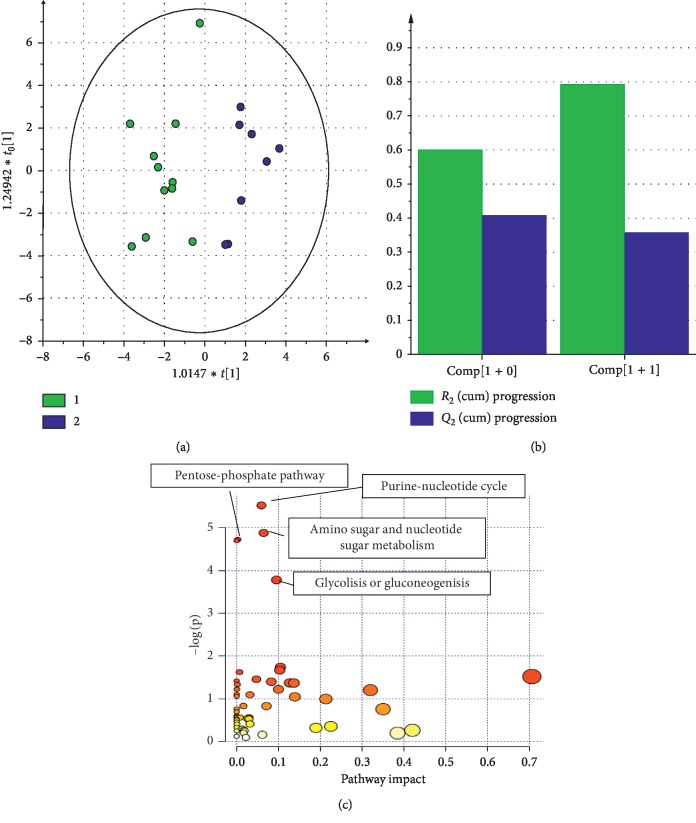
(a) The OPLS-DA model indicated a clear separation between the groups; (b) cross validation (*R*^2 ^= 0.793 *Q*^2^ = 0.358); (c) metabolic hubs mainly affected in CAA patients.

Eleven CAA cases (green dots) and 8 healthy controls (blue dots) underwent metabolomics analysis. The multivariate statistical analysis showed a clear separation between cases and controls. This model was best described by the two principal components, showing *R*^2^ (goodness of fit) = 0.793 and Q^2^ (goodness of prediction) = 0.358; *p* value < 0.05 (Figures 1(a) and 1(b)).

Among the 76 metabolites detected, the CAA group exhibited statistically higher concentrations of 4-hydroxyhydrocinnamic acid, *N*-acetylneuraminic acid, inositol-like (an inositol isomer other than myo-, scyllo-, or chiroinositol), ornithine, putrescine, hypoxanthine, 5-aminopentanoic acid, proline, uracil, lysine, stearic acid, threonine, uric acid, glycine, and phosphoethanolamine with respect to the control group. On the other hand, the CAA group showed statistically lower concentrations of sorbitol, maltose, glucose, xylitol, succinic acid, ethanolamine, lactic acid, palmitic acid, citric acid, urea, 1,2-propanediol, and meso-2,3-butanediol when compared to the controls (Table 2).

The pathway analysis allowed the identification of four distinctive pathways among the CAA patients. The resulted pathways were purine nucleotide cycle, amino sugar and nucleotide sugar metabolism, pentose phosphate pathway, and glycolysis and gluconeogenesis pathways (Table 3).

## 4. Discussion

Chronic apical abscess is an inflammatory disease affecting the periapical tissues and triggered by root canal infection. Among the factors contributing to the development of CAA, a shift in the homeostasis of the oral bacterial towards the dysbiosis of the microbial population is considered important [22]. In this respect, metabolomics enables studies of substances that may turn out to be candidate biomarkers of such condition. In fact, discriminant metabolites constituting the fingerprint of the disease may help to better understand the features sustaining a chronic endodontic infection and to establish better strategies to prevent and deal with these conditions. Recent studies in the fields of metabolomics provided novel information on the pathogenesis of periodontal inflammation, infection, and tissue destruction. All these studies were performed using the saliva, as the biofluid of choice, due to its close contact with the overall oral cavity [23]. Moreover, saliva is easier to handle than blood because it does not clot, thus reducing the number of manipulations required. Saliva is composed of water for 99% of its volume, and the remaining 1% comprises hundreds of small molecules that make it an interesting biofluid for medical testing. The chemical composition of saliva is known to change dramatically in response to different physiological and pathological stimuli [24]. The salivary metabolome of CAA patients in this study presented heavy differences compared to the healthy population, as evidenced by the metabolite list reported in Table 2. The most important biochemical pathways that characterized the saliva from CAA patients were as follows: pentose-phosphate pathways, amino sugar and nucleotide metabolism, glycolysis or gluconeogenesis, and purine nucleotide cycle. Among the metabolites from the abovementioned pathways, some reflect the differences between the two groups of patients. In particular, high levels of 4-hydroxyhydrocinnamic, putrescine, ornithine, hypoxanthine, and phosphoethanolamine and lower levels of ethanolamine and uric acid have already been associated with periodontal inflammatory status in a recent metabolomic study performed in adult patients before and after the removal of supragingival plaque [25]. The authors were eventually able to build a ROC curve by using 4-hydroxyhydrocinnamic and cadaverine that resulted to be highly specific for the identification of severe periodontitis. Furthermore, the production of 4-hydroxyhydrocinnamic was hypothesized to be related to the gut microbiota dysbiosis [26].

Putrescine is a polyamine synthesized by bacteria, such as *E. coli* and *Pseudomonas* species, via two pathways, the decarboxylation of ornithine to putrescine and that of arginine to agmatine, followed by the conversion of agmatine to putrescine and urea [27]. The presence of high level of putrescine could be intended as an adaptation mechanism of the bacteria to counteract a low pH to induce the alkalinisation of the cytosol and the generation of a proton motive force useful for acid stress resistance and for ATP production [28].

The bacterial metabolic fingerprint was also indicated by the presence of a high concentration of phosphoethanolamine, a phosphomonoester metabolite of the phospholipid metabolism, precursor of phospholipid synthesis, and product of phospholipid breakdown. Lipids seem to have an important role in the oral ecosystem and their concentration correlates with the presence of caries [29]. Of interest, reduced levels of ethanolamine by-product in CAA patients has been related to bacterial dysbiosis, since this compound is readily derived from cell membranes that certain bacteria can utilize as a carbon/nitrogen source [30].

Another metabolite related to bacterial overgrowth and/or endogenous tissue necrosis is 5-aminopentanoic acid, whose high concentration is known to be present in patients with chronic periodontitis [31]. Its metabolism seems to be indicative of the activity of anaerobic bacteria such as *Clostridium viridans* [32].

High concentrations of hypoxanthine and uric acid, apparently in contrast, seem to be related to the oxidative stress: hypoxanthine indicates the activation of the purine degradation pathway, a major biochemical source to produce reactive oxygen species (ROS) [33]. High concentration of uric acid, a well-known salivary antioxidant could on the other hand be interpreted as a response of the organism to the ROS production. Redox balance is crucial for the maintenance of normal cellular function and health. The reduction of antioxidants in periodontal diseases has been well documented in the literature [34]. Furthermore, an indirect link with the purine degradation pathway could be hypothesized through the detected decrement of succinate, a Krebs cycle intermediate. The metabolic connection between the purine pathway and the Krebs cycle via the “purine nucleotide cycle” is well known in the biological literature.

## 5. Conclusion

The results of our study should be cautiously interpreted due to the following limitations. The size of our model was too small to draw general extrapolations on the pathophysiological events underlying this complex clinical scenario. The limited number of subjects was due to the need to have all the patients treated in the same way by the same physician in order to reduce all clinical and pharmacological biases. For this reason, all biological samples were collected in a single Dental Clinic at the University Hospital. Nonetheless, the interesting features of this pilot study are the metabolites identified in CAA patients, which seem to be closely related to bacterial catabolism and tissue necrosis, which in turn may be associated with the presence of a sinus tract.

## Figures and Tables

**Table 1:** Clinical and demographic characteristics of patients population.

Patient ID	Gender	Age (years)	Histopathologic diagnosis
1	Female	30	CAA
2	Male	51	CAA
3	Male	33	CAA
4	Female	60	CAA
5	Male	42	CAA
6	Female	68	CAA
7	Male	43	CAA
8	Male	39	CAA
9	Male	60	CAA
10	Male	46	CAA
11	Male	45	CAA
12	Male	37	Control
13	Male	30	Control
14	Male	41	Control
15	Male	38	Control
16	Female	39	Control
17	Female	45	Control
18	Female	56	Control
19	Female	60	Control

**Table 2:** Variable influence in projection (metabolites accumulated or depleted in CAA patients sorted by trend).

VIP: variable influence in projection		
Metabolites	Trend in CAA patients	Score values sorted by trend
4-Hydroxyhydrocinnamic acid	↑	1.90102
*N*-Acetylneuraminic acid	↑	1.70824
Inositol-like	↑	1.35246
Ornithine	↑	1.2671
Putrescine	↑	1.25158
Phosphoethanolamine	↑	1.1257
Hypoxanthine	↑	1.07426
5-Aminopentanoic acid	↑	0.99078
Proline	↑	0.948403
Uracil	↑	0.804598
Lysine	↑	0.764083
Stearic acid	↑	0.698099
Threonine	↑	0.654658
Uric acid	↑	0.630694
Glycine	↑	0.620945
Sorbitol	↓	4.42798
Maltose	↓	3.66992
Glucose	↓	2.58188
Xylitol	↓	1.74627
Succinic acid	↓	1.26194
Ethanolamine	↓	1.17785
Lactic acid	↓	1.13476
Palmitic acid	↓	1.02905
Citric acid	↓	0.995201
Urea	↓	0.886784
1,2-Propanediol	↓	0.774375
meso-2,3-butanediol	↓	0.67866

**Table 3:** Most important pathways based on the metabolic fingerprint.

Pathways mainly affected in CAA patients			
Pathway name	*p* value	False discovery rate (FDR)	Metabolites belonging to the pathway
Purine nucleotide cycle	0,004	0,125	Hypoxanthine, urea, uric acid, glycine, inositol, urea, 1,2-propanediol
Amino sugar and nucleotide sugar metabolism	0,009	0,125	Glucose, pyruvic acid, *N*-acetylneuraminic acid
Pentose phosphate pathway	0,009	0,125	Glucose, pyruvic acid, phosphate, ethanolamine
Glycolysis and gluconeogenesis pathways	0,022	0,242	Lactic acid, pyruvic acid, glucose, citric acid, succinic acid

## Data Availability

The data used to support the findings of this study are available from the corresponding author upon request.
